# The Neurobehavioral Mechanisms Underlying Attitudes Toward People With Mental or Physical Illness

**DOI:** 10.3389/fnbeh.2020.571225

**Published:** 2020-11-12

**Authors:** Won-Gyo Shin, Choong-Wan Woo, Wi Hoon Jung, Hackjin Kim, Tae Young Lee, Jean Decety, Jun Soo Kwon

**Affiliations:** ^1^Department of Psychology, University of Chicago, Chicago, IL, United States; ^2^Institute of Human Behavioral Medicine, Seoul National University College of Medicine, Seoul, South Korea; ^3^Center for Neuroscience Imaging Research, Institute for Basic Science, Suwon, South Korea; ^4^Department of Biomedical Engineering, Sungkyunkwan University, Suwon, South Korea; ^5^Department of Psychology, Daegu University, Gyeongsan, South Korea; ^6^Department of Psychology, Korea University, Seoul, South Korea; ^7^Department of Psychiatry, Seoul National University College of Medicine, Seoul, South Korea; ^8^Department of Psychiatry and Behavioral Neuroscience, University of Chicago, Chicago, IL, United States; ^9^Department of Brain and Cognitive Sciences, Seoul National University, Seoul, South Korea

**Keywords:** fMRI, mental illness, stigma, prejudice, empathic concern

## Abstract

Social factors play a significant role in the health outcomes of those struggling with mental or physical health issues. People with mental illness experience more social stigmatization and receive less concern for their welfare than do those with physical illness. However, the cognitive and neural mechanisms by which such a bias in attitude arises remain unclear. This functional MRI study examined whether a lack of self-other similarity during mental state attribution affects perceivers’ theory of mind and, subsequently, how they value a patient’s welfare. During scanning, participants were asked to respond to an expression of caring and sympathetic concern from either their own perspective or while adopting the perspective of patients labeled physically ill or mentally ill. Participants reported that physically ill patients would share their affective responses to the situations, but mentally ill patients would not. Furthermore, mentalizing about physically ill patients was associated with increased activity in the ventromedial prefrontal cortex (vmPFC), a critical region for empathic concern and value-based decisions. In contrast, mentalizing about mentally ill patients preferentially engaged the dorsal anterior cingulate cortex (dACC) and anterior insula, regions previously implicated in empathic distress, in which activity correlated with individual differences in prejudice control. The findings indicate that a lack of perceived self-other similarity poses a challenge to the theory of mind and thus requires greater cognitive resources and neural computations. This might give rise to stereotyped beliefs about and prejudice against the mentally ill and failure to respond with appropriate empathy and care.

## Introduction

Mental illness accounts for one of the largest growing categories of the global burden of disease (Collins et al., 2011; Steel et al., 2014). Yet despite its considerable burden and its devastating effects on individuals, families, and communities, the resources allocated to caring for the mentally ill are inadequate (Vigo et al., 2016). The pervasive stigma and discrimination contribute to this imbalance, preventing appropriate care and treatment from reaching those with mental illness (Henderson et al., 2013; Corrigan et al., 2014; Vigo et al., 2019). Stigma is among the major barriers to seeking and engaging in care, adherence to treatment, and interpersonal relationships (Sartorius, 2007; Clement et al., 2015; Stangl et al., 2019), which have wide-ranging pernicious effects on health (Chesney et al., 2014; Walker et al., 2015; Major et al., 2017). This situation leads to a fundamental question about why it is common to stigmatize the mentally ill, although we often sympathize with the physically ill.

Current theories regarding stigma include social cognitive models (Corrigan, 2000) and sociological models (Link and Phelan, 2001). In social cognitive models, signals such as psychiatric labels or symptoms yield negative beliefs. These stereotypes lead to prejudice (agreement with stereotyped beliefs, or negative affective responses towards a group) and discrimination (a behavioral consequence of prejudice). Sociological theories suggest that stigma occurs when human differences are socially selected for salience and labeled. Importantly, labeling implies a separation of “us” from “them,” and this separation easily leads to the belief that “they” are fundamentally different from “us” (Link and Phelan, 2001; Rüsch et al., 2005). However, it remains unclear as to the mechanism by which a psychiatric label shapes stigma-related beliefs and any particular attribute and underlying processes by which the mentally ill are believed to be distinctly different from ourselves. Notably, unlike some stigmatized markers such as race, obesity, or physical disfigurement, mentally ill patients usually do not have physical attributes that mark them as different (Jones et al., 1984; Corrigan, 2000; Major et al., 2017). Thus, an alternative explanation for the difference may be related to assumed dissimilarity in subjective experiences, such as feelings, thoughts, and desires.

Biased attitudes operate implicitly, influencing behaviors without awareness, and their behavioral effects are often regulated by social norms (Devine, 1989; Amodio, 2014). A social neuroscience approach contributes to elucidating how and under what conditions people’s attitudes are biased, expressed, and controlled (Krendl et al., 2006, 2012). Here, we examine whether perceivers assume that, unlike the physically ill, the mentally ill would experience different mental states in the same situations and thus rely on a different set of social cognitive processes when making inferences about their mental states. Specifically, we framed our question in the context of valuing the welfare of patients because the primary issue surrounding mental illness is the lack of public support and health care services (Vigo et al., 2019). Addressing this issue has the potential to improve existing theoretical frameworks of stigma. Further, it may provide a step towards new approaches to the multidisciplinary analysis of health-related stigmas and the development of more tailored public health interventions.

From a social cognition standpoint, humans are guided by internal states and survival depends on interactions with others. The ability to identify and understand others’ mental states plays a pivotal role in interpersonal relationships and empathic responses (Kovács et al., 2010; Shamay-Tsoory, 2011; Heyes and Frith, 2014). When inferring others’ mental states, perceivers put themselves into another’s perspective and imagine how that person feels or thinks. Various lines of evidence suggest that adopting the perspective of another person, particularly someone from a different social group, is cognitively demanding and hence requires higher demands on executive resources (Lamm et al., 2010; Decety and Cowell, 2014). For example, in addition to brain areas involved in theory of mind, studies investigating third-person perspective taking have found increased activation in prefrontal areas associated with executive function, working memory, and inhibition (Ruby and Decety, 2003, 2004). In this respect, it might be that taking the perspectives of those with mental illness poses a challenge to the theory of mind and requires more effortful cognitive processes, thus placing a heavy load on executive functioning.

Social cognition can also be viewed as a balance in the extent to which shared representations between self and other are activated and managed (Decety and Sommerville, 2003; Southgate, 2020). These representations provide a basic mechanism for social cognition and account for the ability to identify with others (Ruby and Decety, 2003, 2004). In particular, shared mental representations can be expected when people use their self-knowledge, experiences, and mental states to infer others’ mental states. Importantly, such self-referential processing is useful to the extent that it can be assumed that others think and feel similarly to oneself (Mitchell et al., 2005). This notion is further supported by a ventral-dorsal gradient in the medial prefrontal cortex (mPFC) for mentalizing about similar and dissimilar others (Mitchell et al., 2006; Jenkins et al., 2008; Tamir and Mitchell, 2010). These studies suggest that prejudice arises from the perceivers’ assumption that the target’s mental states are substantially different from their own, hence mentalizing in a non-self-referential way. However, it is unknown whether the self-referential account of social cognition can be extended to other domains, such as biased attitudes toward mental and physical illness.

Furthermore, some forms of prosocial behavior, such as caring and helping, are motivated primarily by empathic concern, the tendency to experience feelings of sympathy or compassion for others (Williams et al., 2014; Decety et al., 2016). Functional neuroimaging studies have shown that empathic concern motivates costly altruism and charitable donations. These associations are predicted by the hemodynamic response in the ventromedial prefrontal cortex (vmPFC; FeldmanHall et al., 2015; Ashar et al., 2017). Activity in the vmPFC has been associated with the computation of stimulus values during prosocial choice (Hare et al., 2010; Sul et al., 2015) and plays a role in empathic concern and caregiving (Parsons et al., 2013; Decety and Cowell, 2014). Neurological patients with damage to the vmPFC do not differ in the theory of mind but experience less empathy and motivation to care (Beadle et al., 2018). Together, these studies provide evidence that the vmPFC is critical for empathic concern toward individuals suffering or in need. However, empathy is flexible (Zaki and Ochsner, 2012; Decety et al., 2016) and is modulated by situational context (Cheng et al., 2017), social factors such as the target’s stigmatized status (Decety et al., 2010; Azevedo et al., 2014), attitudes toward ingroup and outgroup members (Hein et al., 2010), and perceived closeness with the target (Cheng et al., 2010). Notably, empathic concern increases or decreases depending on whether the perceived welfare of a person in need is valued more positively or negatively (Batson, 2011). Some studies have reported that higher valuing of the other’s welfare leads to greater empathic concern, which subsequently results in better assistance (Batson et al., 2007).

Encountering another person suffering can elicit empathic concern, but it can also lead to personal distress in the form of empathic distress. This is an aversive self-focused emotional response that often results in withdrawal behavior and avoidance to protect oneself from negative feelings, thereby reducing the likelihood of prosocial behavior (Decety and Lamm, 2009). Empirical evidence suggests that empathic distress and empathic concern are supported by distinct brain mechanisms (Ashar et al., 2017). Functional neuroimaging studies have shown that neural processing in the anterior cingulate cortex (ACC) and anterior insula is associated with personal distress (Lawrence et al., 2006; Cheetham et al., 2009; Fan et al., 2011). In particular, the anterior insula, through its connection with the ACC and prefrontal cortex (PFC), contributes to the subjective experience of negative affect as part of a prejudicial response and the engagement of prejudice control (Amodio, 2014). Given these considerations, a negative attitude toward people with mental illness may trigger an aversive emotional response associated with activity in the ACC and anterior insula, which in turn will lead to reduced caring motivation for their welfare compared to physically ill patients. However, at present, it remains underexplored how empathic response and caring motivation is affected by a patient’s group label of mentally ill or physically ill.

Taken together, the aforementioned studies converge in suggesting that attitudes toward those with physical or mental illness may depend on whether a self-other similarity can be assumed in subjective experiences such as feelings and thoughts, which may in turn influence the value placed on the patients’ welfare and empathic responses to them. To test these hypotheses, we used functional magnetic resonance imaging (fMRI) while participants were asked to infer the mental states of patients labeled physically ill or mentally ill in response to messages of caring and sympathetic concern and to indicate how they themselves would respond to the same messages. We expected to find that mentalizing about physically ill patients would be associated with empathic concern and activity in the vmPFC while mentalizing about mentally ill patients would be associated with prejudice-related responses and activity in the ACC and anterior insula. Examining these questions has important theoretical and societal significance for public health issues, such as stigma and lack of social support, and could inform intervention development, research, and policy. It will also improve our understanding of human social cognition, such as empathy, perception of self-other distance, and mind-reading, and their relationship to these public health issues.

## Materials and Methods

### Participants

Healthy adults aged 19–35 were first recruited *via* an Internet advertisement. They were then screened through face-to-face interviews and the Symptom Checklist-90-R (SCL-90-R; Derogatis and Cleary, 1977; Kim and Kim, 1984). A total of 42 right-handed individuals participated in the fMRI experiment. Data from two participants were excluded from the analysis due to excessive head motion greater than 3 mm during scanning. The remaining 40 participants (30 females, 10 males; mean and SD of age, 22.8 ± 2.09) had no history of brain injury or neurological or psychiatric disorders, first- or second-degree relatives with a history of a psychotic disorder, medical or psychiatric disorders currently in treatment, or contraindications for MR scanning. The study was approved by the Institutional Review Board of Seoul National University Hospital, and written informed consent was obtained from each subject.

### Experimental Paradigm

**Figure 1:**
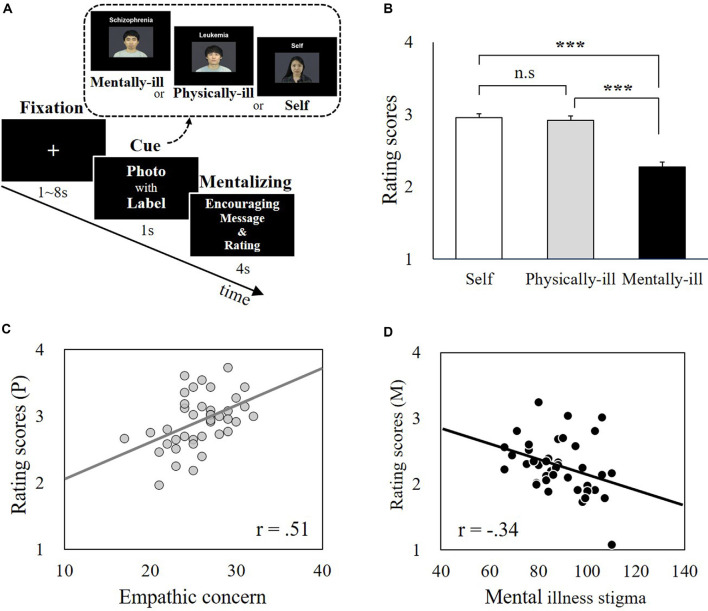
Experimental paradigm and behavioral data. **(A)** The supportive message-judging task during functional magnetic resonance imaging (fMRI) scanning. Participants (*N* = 40) were asked to infer the mental states of patients labeled with mental illnesses (i.e., mental-illness-trials) or physical illnesses (i.e., physical-illness-trials) in response to supportive messages and to indicate their own responses (i.e., self-trials). **(B)** There was no significant difference between self-trials and physical-illness-trials (*t* = 1.01, *p* = 0.32), but the mentally ill were expected to be less encouraged by supportive messages than the self (*t* = 8.36, *p* < 0.001) and the physically ill (*t* = 7.59, *p* < 0.001). **(C)** Correlation between participants’ empathic concern level and their prediction of the response of the physically ill (*p* = 0.001). **(D)** Correlation between participants’ mental illness prejudice level (i.e., negative attitude) and their prediction of the responses of the mentally ill (*p* = 0.03). ***p < 0.001; P, physical-illness-trials; M, mental-illness-trials. n.s, not significant.

During the fMRI scanning, participants were asked to infer the mental states of patients categorized as being either physically ill or mentally ill and to predict the patients’ responses to caring in the form of supportive messages (Figure 1A). The participants were also asked to indicate how they would respond to the same set of messages. To prevent the participants from responding randomly and to increase engagement in the task, participants were told that supportive messages would be sent to the patients based on the study’s findings. This encouraged participants to predict the actual responses of these patients as accurately as possible. Trials were divided into priming and judgment phases. Each trial began with the presentation of one of three priming stimuli: (i) a labeled photo of a physically ill target (physical-illness-trials); (ii) a labeled photo of a mentally ill target (mental-illness-trials); or (iii) a labeled photo of the participant (self-trials). The priming stimuli appeared above a four-point response scale corresponding to different levels of encouragement that a recipient might feel (anchored by “1 = definitely not encouraged” and “4 = definitely encouraged”). After 1 s, a supportive message appeared between the priming image and the response scale. The participants were asked to guess how much the target would feel encouraged by each of the 48 messages. For the trials utilizing the participant’s own picture, they were asked to report how much they would be encouraged by the messages. The labeled photo, supportive message, and scale remained onscreen together for 4 s. A total of three scanning runs were performed, with each run comprised of 48 trials (16 self-trials; 16 physical-illness-trials; 16 mental-illness-trials), resulting in a total of 144 trials (48 self-trials; 48 physical-illness-trials; 48 mental-illness-trials). To optimize the estimation of the event-related fMRI response, all stimuli were presented in a pseudorandomized order and separated by a variable inter-stimulus interval created by the genetic algorithm (500–7,900 ms; Wager and Nichols, 2003) and the order of runs was counterbalanced across participants. Outside of the scanner, participants completed a questionnaire of empathy and prejudice.

### Stimuli

#### Socially Supportive Messages

Supportive messages were employed as a representative form of prosocial behavior, since they are a simple way of showing support and concern and, despite their concise form, can have meaningful positive effects on the listener (Coulson et al., 2007; Rains et al., 2016). A total of 48 messages with a rating score over four points (anchored by “1 = definitely not encouraged” and “7 = definitely encouraged”) were selected from a previously conducted behavioral pilot study (*n* = 56). Based on previous research using social support messages (Mo and Coulson, 2008; Evans et al., 2012), the messages included content related to emotional support (e.g., *You are in my heart and mind*), esteem support (e.g., *You are stronger than you know*), or network support (e.g., *Anytime you want to talk to anyone of us, feel free to let us know*).

#### Target Photos

A total of 32 photos with neutral facial expressions were drawn from a previously validated set (Lee et al., 2013). Among these photos, 16 were labeled as portraying mental illness, and 16 others were labeled as portraying physical illness. There were no differences in valence, arousal, sex, or attractiveness between the photos chosen for the physically ill and mentally ill patients. The assignment of the target patients to the type of illness was counterbalanced across participants.

#### Self-Photos

The photos of participants were taken during their eligibility screening 2 weeks before the fMRI scanning. These photos were edited to the same size and resolution as those of the photos of targets.

#### Specific Mental and Physical Illnesses

From a list of words related to mental and physical illnesses, specific illnesses were selected according to representativeness and familiarity. Four psychiatric disorders (schizophrenia, depression, paranoid disorder, and OCD) and four physical diseases (leukemia, cancer, diabetes, and arthritis) were selected and classified. The word “cancer” was changed to “thyroid cancer” to match the average number of syllables between two categories. The severity between four mental illnesses and four physical illnesses was balanced.

### Behavioral Questionnaires and Task

Before fMRI scanning, participants completed a set of self-reported dispositional measures. The Interpersonal Reactivity Index (IRI) was administered to assess empathy (Davis, 1983; Kang et al., 2009). Three subscales of the IRI (i.e., empathic concern, perspective-taking, and personal distress) were used. After the scanning session, participants were given questionnaires to assess their explicit prejudice toward the mentally ill using the Korean version (Lee et al., 1996) of the Community Attitudes Toward the Mentally Ill Scale (CAMI; Taylor and Dear, 1981). To assess participants’ motivation to respond without prejudice, we used a version of the Internal and External Motivation to Respond Without Prejudice scale (Plant and Devine, 1998), which was translated to Korean using the forward-backward translation process. The implicit Association Test (IAT; Greenwald et al., 1998) was used to measure implicit attitudes toward the mentally ill.

### Image Acquisition

Blood-oxygenation-level-dependent (BOLD) signal was acquired using a 3T Siemens Trio MRI scanner (Siemens Healthcare, Erlangen, Germany) using T2*-weighted gradient echo planar imaging [repetition time (TR) = 2,000 ms; echo time (TE) = 30 ms; field of view (FOV) = 220 mm; flip angle = 80°; slice thickness = 3.4 mm; matrix = 64 × 64]. T1 anatomical volume reference images were also acquired (TR = 2,400 ms; TE = 2.19 ms; FOV = 272 mm; flip angle = 8°; slice thickness = 0.85 mm; matrix = 320 × 320). Field map images for distortion correction were also collected (TR = 425 ms; TE1 = 4.92 ms; TE2 = 7.38 ms; FOV = 220 mm; slice thickness = 3.4 mm; matrix = 64 × 64). Foam pads were used to minimize head motion.

### Behavioral Data Analysis

Paired *t*-tests were used to compare the encouragement scores (ratings of how encouraged a recipient would feel) of supportive messages in the three conditions: self-trials, physical-illness-trials, and mental-illness-trials. Significance levels were set at *p*-values less than 0.05, divided by the number of comparisons (*p* < 0.05, Bonferroni corrected; that is, *p* = 0.05/3 = 0.017). To identify individual dispositions preferentially related to the estimated encouragement scores of the physically ill and the mentally ill, Pearson correlation analysis was used. A stepwise regression model was performed to identify the most influential individual dispositions in predicting the expected value difference between physical-illness-trials and mental-illness-trials. Delta values were determined by computing differences between rating scores in physical-illness-trials and mental-illness-trials, such that positive values reflected a higher degree of expected encouragement felt by the physically ill than for the mentally ill (i.e., difference score_physical–mental_ = rating score__physical-illness-trials_ − rating score__mental-illness-trials_). All dispositional measures were entered into the regression model.

### Functional Imaging Data Analysis

fMRI data were processed and analyzed with the Statistical Parametric Mapping (SPM) software (version 12; MathWorks, Inc). The first three images were discarded to avoid unstable magnetic artifacts. First, functional data were time-corrected for differences in acquisition time between slices for each whole-brain volume. Then data were realigned to correct for head movement and unwarped using the field map. The mean realigned and the unwarped image was normalized to the EPI template in the Montreal Neurological Institute (MNI) space and was resampled into 2-mm^3^ voxels. Normalized data were then spatially smoothed with an 8 mm full-width-at-half-maximum (FWHM) by using a Gaussian kernel. Functional activation analyses were performed by using the general linear model (GLM) with hemodynamic response function (HRF) for each of the conditions (i.e., self-trials; physical-illness-trials; mental-illness-trials). Residual effects of head motion were corrected for by including the six estimated motion parameters (x, y, z, roll, pitch, and yaw), their mean-centered squares, their derivatives, and the squared derivative for each run (total 24 columns). To assess random effects at the group level, contrast images generated at the individual level were entered into one-sample *t-*tests. To compare differences in brain regions engaged preferentially during mentalizing about physically ill patients and mentally ill patients, we calculated the first-level contrast images for the physical-illness-trials vs. mental-illness-trials and the mental-illness-trials vs. physical-illness-trials contrasts and then entered them into one-sample *t-tests*. Next, to identify brain regions in which the hemodynamic activity correlated with the value that each participant placed on the supportive message in each condition, the second GLM analysis with parametric modulators (i.e., encouragement scores of each supportive message) with 24 head movement parameters was conducted. Following the behavioral results of the similar rating patterns between self-trials and physical-illness-trials (Figure 1B), our interest was to observe whether brain regions activated in self-trials were similarly engaged during mentalizing about the physically ill. Thus, we calculated the first-level contrast images for the self-trials vs. baseline, the physical-illness-trials vs. baseline, and the mental-illness-trials vs. baseline contrasts, and then entered them into one-sample *t-tests*. Groupwise contrasts were thresholded at *p* < 0.05, whole-brain familywise error (FWE) corrected or small volume FWE corrected based on cluster extent with a primary threshold of *p* < 0.001 or *p* < 0.0001, respectively. Regions-of-interest (ROIs) were identified from spheres with a radius of 5 mm around the peak voxel for each brain region showing significant activation in the whole-brain analysis. The mean percent BOLD signal change was extracted from the ROIs using the MarsBaR toolbox^1^. To capture individual differences in the signal changes found in those two contrasts (i.e., mental-illness-trials vs. physical-illness-trials and physical-illness-trials vs. mental-illness-trials contrasts), the ROIs were then correlated with dispositional measures. Bonferroni corrections were applied to avoid the problem of multiple comparisons.

## Results

### Behavioral Data

Participants rated that mentally ill patients would not feel similar levels of encouragement in response to supportive messages, as they themselves would (*t*_(39)_ = 8.36, *p* < 0.001, Bonferroni-corrected *p* = 0.05/*3* = 0.017) and physically ill patients would (*t*_(39)_ = 7.59, *p* < 0.001, Bonferroni-corrected *p* = 0.05/*3* = 0.017). There was no significant difference between self-trials and physical-illness-trials (*t*_(39)_ = 1.01, *p* = 0.32; Figure 1B). The estimated encouragement scores of the physically ill and the mentally ill were correlated with dispositional measures including empathy and mental illness prejudice. The estimated encouragement scores of the physically ill were associated with empathic concern (*r*_(39)_ = 0.51, *p* = 0.001, Bonferroni-corrected *p* = 0.05/*3* = 0.017; Figure 1C) and perspective taking (*r*_(39)_ = 0.37, *p* = 0.02, uncorrected), but not with personal distress (*r*_(39)_ = −0.19, *p* = 0.24). In contrast, the estimated encouragement scores of the mentally ill were not linked to any disposition in empathic concern (*r*_(39)_ = −0.24, *p* = 0.13), perspective taking (*r*_(39)_ = −0.25, *p* = 0.11), or personal distress (*r*_(39)_ = 0.04, *p* = 0.80). Instead, participants’ predictions of the responses from the mentally ill were associated with the levels of explicit (*r*_(39)_ = −0.34, *p* = 0.03, uncorrected) and implicit (*r*_(39)_ = −0.36, *p* = 0.03, uncorrected) prejudice toward the mentally ill (Figure 1D).

The stepwise regression analysis identified the most influential individual dispositions in predicting the expected value difference of prosocial behavior between physical-illness-trials and mental-illness-trials. The final model included three predictors: empathic concern, mental illness prejudice, and motivation to respond without prejudice. Empathic concern accounted for 32% of the variance, while empathic concern and mental illness prejudice accounted for 47%, and empathic concern, mental illness prejudice_,_ and motivation to respond without prejudice accounted for 53% of the difference score_(physical–mental)_ which was significant (*F*_(36)_ = 13.56, *p* < 0.001; Table 1).

**Table 1:** The best model of behavioral predictors.

	*R^2^*	*F*-value	Sig. *F* change
**Model 1**			
Empathic concern	0.32	17.57***	*p* < 0.001
**Model 2**			
Empathic concern, Mental illness prejudice	0.47	16.13***	*p* = 0.003
**Model 3**			
Empathic concern, Mental illness prejudice, Motivation to respond without prejudice	0.53	13.56***	*p* = 0.032

A stepwise regression model examining predictors for the expected value difference of prosocial behavior between physical-illness-trials and mental-illness-trials. Durbin-Watson = 2.10; Variance Inflation Factor (VIF) < 2. ***p < 0.001.

### Functional MRI Data

**Figure 2:**
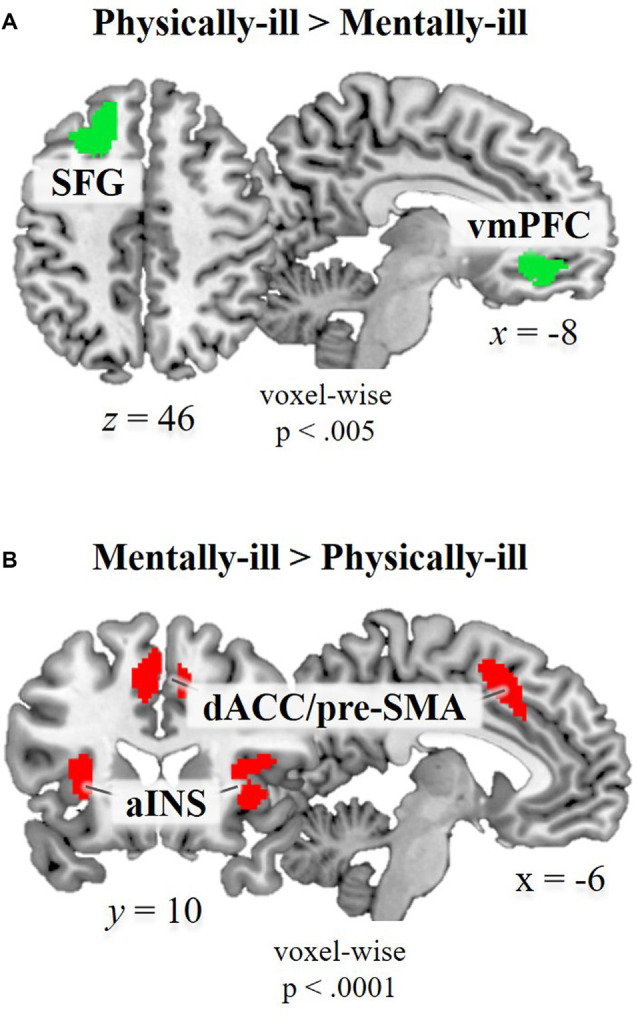
Functional magnetic resonance imaging (fMRI) data. Brain regions showing significant hemodynamic increases **(A)** in physical-illness-trials vs. mental-illness-trials at *p* < 0.5, SV−familywise error (FWE) corrected based on cluster extent with a primary threshold of *p* < 0.001; for display at *p* < 0.005 and **(B)** in mental-illness-trials vs. physical-illness-trials at *p* < 0.5, FWE corrected based on cluster extent with a primary threshold of *p* < 0.0001. SFG, superior frontal gyrus; vmPFC, ventral medial prefrontal cortex; aINS, anterior insula; dACC, dorsal anterior cingulate cortex; pre-SMA, pre-supplementary motor area; SV−FWE, small-volume family-wise error.

Whole brain analysis contrasting physical-illness-trials vs. mental-illness-trials during mentalizing about the patients revealed increased activity in the vmPFC (*t*_(39)_ = 4.22, *p* < 0.001) and superior frontal gyrus (SFG; *t*_(39)_ = 4.19, *p* < 0.001; Figure 2A). The reverse contrast (i.e., mental-illness-trials vs. physical-illness-trials) was associated with significantly increased activation in the anterior insula (left, *t*_(39)_ = 7.07, *p* < 0.0001; right, *t*_(39)_ = 5.68, *p* < 0.0001), dorsal anterior cingulate cortex (dACC; left, *t*_(39)_ = 6.37, *p* < 0.0001; right, *t*_(39)_ = 5.94, *p* < 0.0001), thalamus (*t*_(39)_ = 7.10, *p* < 0.0001), precuneus (left, *t*_(39)_ = 5.46, *p* < 0.0001; right, *t*_(39)_ = 5.68, *p* < 0.0001), and left lingual gyrus (*t*_(39)_ = 7.87, *p* < 0.0001; Figure 2B).

**Figure 3:**
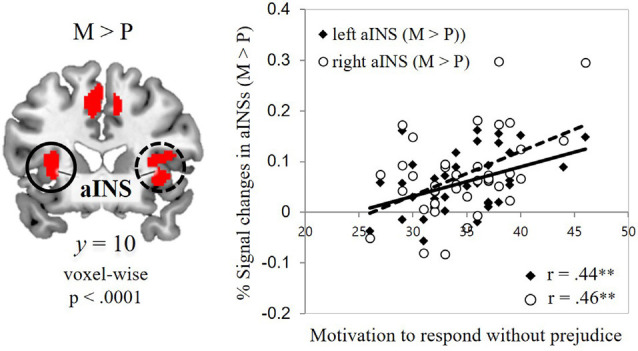
Associations between self-report and fMRI data. Correlations of the signal change in the mental-illness-trials vs. physical-illness-trials in the bilateral anterior insula with individual differences in motivation to respond without prejudice. aINS, anterior insula; P, physical-illness-trials; M, mental-illness-trials. **p < 0.01.

To capture individual differences in the signal changes found in those two contrasts, ROIs were defined from the contrast maps (two ROIs for physical-illness-trials vs. mental-illness-trials contrast; eight ROIs for mental-illness-trials vs. physical-illness-trials contrast). Hemodynamic activity in these ROIs was then correlated with dispositional measures of empathy and prejudice. Motivation to behave without prejudice was significantly associated with the signal change in the mental vs. physical contrast in the left anterior insula (*r* = 0.44, *p* = 0.005, Bonferroni-corrected *p* = 0.05/8 = 0.006) and right anterior insula (*r* = 0.46, *p* = 0.003, Bonferroni-corrected *p* = 0.05/8 = 0.006; Figure 3).

**Figure 4:**
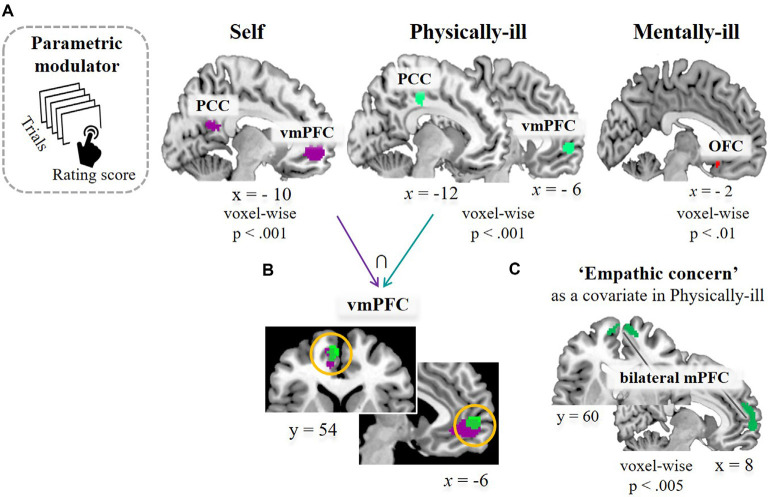
Task-related brain activation. **(A)** Results of a parametric modulation analysis using subject-specific value parameters (i.e., encouragement scores of each supportive message) in self-trials (left, at *p* < 0.5, FWE corrected based on cluster extent with a primary threshold of *p* < 0.001), in physical-illness-trials (middle, at *p* < 0.5, SV−FWE corrected based on cluster extent with a primary threshold of *p* < 0.001), and in mental-illness-trials (right, at *p* < 0.01, uncorrected for display). **(B)** Shared neural responses in vmPFC between self-trials and physical-illness-trials. **(C)** Response in mPFC in physical-illness-trials when the empathic concern scores were entered as a covariate (at *p* < 0.005, uncorrected for display). PCC, posterior cingulate cortex; vmPFC, ventral medial prefrontal cortex; OFC, orbitofrontal cortex; mPFC, medial prefrontal cortex; SV−FWE, small-volume family-wise error.

Next, the second GLM analysis with parametric modulators (i.e., encouragement scores of each supportive message) was performed. Following the behavioral results of the similar rating patterns between self-trials and physical-illness-trials (Figure 1B), we were interested in observing whether the brain regions activated in self-trials were similarly engaged during mentalizing about the physically ill. Increased activity within the vmPFC was associated with higher ratings of the supportive messages in the self-trials (*t*_(39)_ = 4.72, *p* < 0.001; Figure 4A, left). The vmPFC was engaged in computing the estimated impact of the supportive messages during mentalizing about physically ill patients (*t*_(39)_ = 4.22, *p* < 0.001; Figure 4A, middle), and the activity within the vmPFC overlapped during self-trials and physical-illness-trials (Figure 4B). However, such associations were not observed even at a lenient threshold (*p* < 0.01) when inferring the mental states of mentally ill patients (Figure 4A, right). Finally, when dispositional empathic concern scores were entered as a covariate, activity in the mPFC was found to be increased in physical-illness-trials (at *p* < 0.005, uncorrected; Figure 4C).

## Discussion

For those suffering from mental or physical health issues, therapy is not just found in a hospital with a specialist but also in words of encouragement and supportive social relationships in their daily lives. The current study, combining attitudinal, behavioral, and functional neuroimaging measures, examined why people usually sympathize with physically ill patients but often stigmatize those who are mentally ill, even though both types of patients suffer from illness. Here, we identified distinct cognitive and neural mechanisms by which such a bias in attitudes toward those with mental and physical illness arises.

Consistent with our hypothesis, behavioral data showed that participants believed that physically ill patients would be encouraged by supportive messages to the same extent that they themselves would and that the physically ill patients would feel more encouraged than mentally ill patients. Additionally, participants who were high in dispositional empathic concern predicted a higher level of encouragement felt by physically ill patients. However, such a link was missing when predicting the responses of the mentally ill. At the neural level, the hemodynamic response in the vmPFC was more engaged when mentalizing about physically ill patients than mentally ill patients. These neural and behavioral findings provide an important clue as to why caring behavior is more rarely directed toward the mentally ill than to the physically ill. The vmPFC plays a key role in representing subjective value (Levy and Glimcher, 2012; Roy et al., 2012; D’Argembeau, 2013; Sul et al., 2015; Delgado et al., 2016) and promoting a caring motivation (Parsons et al., 2013; Decety and Cowell, 2014). Furthermore, the literature suggests that perceiving another in need and valuing the other’s welfare are sufficient conditions for the perceiver to feel empathic concern (Batson et al., 2007; Batson, 2011), which is a primary driver for prosocial behavior such as caregiving and helping (Williams et al., 2014; Decety et al., 2016). In light of such findings, the relatively less-valued welfare of the mentally ill observed here is not a sufficient condition for the perceiver to feel empathic concern, which will lead to reduced caring motivation for the welfare of the mentally ill compared to the physically ill.

Furthermore, a more ventral part of the mPFC is implicated in self-referential processing (van der Meer et al., 2010; Qin and Northoff, 2011), which is often observed when perceivers infer that others feel and think similarly to themselves (Mitchell et al., 2006; Jenkins et al., 2008; Tamir and Mitchell, 2010). In the current study, we found that the vmPFC and posterior cingulate cortex (PCC) varied parametrically with the encouragement ratings in mentalizing about both the self and the physically ill. Mentalizing about those two conditions was further characterized by overlapping self-other activity within the vmPFC. This finding may reflect the perceivers’ recognition that the physically ill are governed by the same types of mental states as themselves, and thus initiate more spontaneous use of their minds as a proxy for those of the physically ill. These data suggest that the self-referential or simulation accounts of social cognition can be extended to the biased attitudes toward those with mental or physical health issues. Together, our results suggest that valuing others’ welfare and shaping motivation to care for them may rely more heavily on perceived self-other similarity in mental states than in bodily states. This idea can improve our current approaches to understanding human empathy and prosocial behavior such as caring and helping. Further, it sheds some light on their relationship with different domains of self-other similarity.

As expected, the participants rated that mentally ill patients would not experience similar levels of encouragement in response to supportive messages, as they and physically ill patients would do. Also, participants high in prejudice against mental illness predicted a lower level of encouragement felt by the mentally ill. At the neural level, a separate set of regions was preferentially engaged during mentalizing about mentally ill patients compared with physically ill patients. These brain regions included the dACC, anterior insula, thalamus, precuneus, and lingual gyrus. The exact contribution of each brain region to the performance of this complex, the high-level cognitive task remains an open question. However, we expected to find that mentalizing about mentally ill patients would be associated with prejudice-related responses and increased activity in the ACC and anterior insula based on previous findings showing that activity in the ACC and anterior insula is associated with an aversive state of personal distress (Lawrence et al., 2006; Cheetham et al., 2009; Fan et al., 2011). Thus, activity in the ACC and anterior insula in our study may reflect participants’ negative emotional responses toward mentally ill patients, and thus adopting the patients’ perspective is likely to induce personal distress.

Interestingly, individual dispositions in motivation to respond with prejudice were associated with increased activation in the anterior insula. Recent work regarding prejudice has also suggested that the anterior insula, through its connections with the ACC and PFC, is considered to facilitate the detection of prejudiced affect and control prejudiced behavior (Amodio, 2014). Participants in this study were primarily young adults drawn from a student population. In these younger and educated individuals, stigmatizing attitudes, manifesting covertly and unconsciously, are countered more often by their egalitarian beliefs and social norms than those of older people and those with low education (Herek, 1999; Decety et al., 2010; Amodio, 2014). Given these considerations, greater activity in the anterior insula observed here may reflect participants’ efforts to restrain expressing unwanted bias toward mental illness, possibly guided by normative expectations about diversity and equality.

Furthermore, evidence has shown that the anterior insular provides an interface with the dorsal parts of the ACC and mPFC, which are involved in cognitively demanding tasks (Etkin et al., 2011; Uddin, 2015), high-level cognitive control, and attentional processing (Menon and Uddin, 2010), and decision under risk and uncertainty (Paulus et al., 2003; Huettel et al., 2006). In this context, our data suggest that the self-other overlap in terms of mental states is hardly assumed in the case of the mentally ill. This may demand heavy cognitive processing due to difficulty in imagination, and thus require greater neural computations and cognitive resources, including working memory and attention. Together, the findings of prejudice-related responses and increased activity in the ACC and anterior insula may help to explain why caregiving behaviors are less directed toward mentally ill patients than to physically ill patients.

The present study sheds some light on the nature and neurobiological foundation of mental illness-related stigma. The current theories suggest that the first step toward stigmatization is to distinguish and label human differences that matter socially (Link and Phelan, 2001; Rüsch et al., 2005) or that signaling events including the label of mental illness yield stereotypes about the mentally ill (e.g., dangerousness/unpredictability), which lead to behavioral reactions (e.g., fear) or discrimination (e.g., avoidance; Corrigan, 2000; Corrigan and Watson, 2002). However, it remains largely unknown as to how a psychiatric label shapes stigma-related beliefs. Further, it is unclear which attribute is socially singled out and prompts people to believe that, unlike the physically ill, the mentally ill are different from themselves. Here, we show that the assumed dissimilarity in mental states (e.g., feelings, beliefs, and intentions) offers a likely answer to both questions. The label of mental illness seems to lead others to assume that the mental states of psychiatric patients do not correspond with their own, which results in the idea that the mentally ill do not represent the world as a comprehensible reality. This may in turn yield stereotyped beliefs about and prejudice against those with mental illness that lead to discriminatory behavior. This explanation may help advance the current theories regarding mental illness stigma and have important implications for the development of new interventions aimed at reducing the stigmatization process or mitigating its negative consequences on health outcomes.

This study also reveals that many people believe care and support for mentally ill patients is not helpful. Contrary to this biased belief, empirical studies have demonstrated that perceived social support positively influences patients’ progress by reducing associated symptoms, increasing psychological stability and quality of life, and improving attitudes toward treatment (Hansson, 2006; Thoits, 2011; Naslund et al., 2016). In contrast, low social support and weak social bonds have been shown to prevent the mentally ill from seeking treatment, which in turn decreases the likelihood of their recovery from their disability (Corrigan et al., 2014). Thus, it is crucial for people to realize the gap between their belief and reality to prevent the mentally ill from being overlooked or ignored by public support and health care initiatives. Such public concern for the welfare of the mentally ill as well as the physically ill will help improve and encourage the patients’ health and health care.

Several limitations of the current study need to be considered. First, although patients with mental illness are usually more rejected from society than those with physical illness, stigmatizing attitudes are not confined only to mental illness. Some types of physical health issues including HIV, obesity, or cerebral palsy experience stigma and social avoidance from others as well. Therefore, evaluating the stigma attached to each illness in terms of its underlying mechanisms and consequences would lead to a better understanding of mental illness stigma as a whole. Second, the demographic characteristics of the sample may limit the generalizability of the findings. Most of the participants were primarily young adults drawn from the student population. These younger and better-educated individuals may manifest lower levels of mental illness stigma than older or less educated people. Moreover, although gender has been found to influence prejudice towards mental illness, we did not analyze the effect because of the insufficient sample size. Thus, the inclusion of various age groups and educational levels, in addition to an examination of gender differences in a larger sample, would provide a more comprehensive view of biased attitudes towards the mentally ill and the physically ill.

In summary, the present study provides the first evidence of differential neural encoding of attitudes toward people with mental and physical illnesses. The findings suggest that distinct representations of different domains of similarity/dissimilarity from the self serve as an important anchor point from which to value the welfare of others and respond with empathy and care. In this respect, mental illness seems to require greater cognitive resources and neural computations due to difficulty in mental state inferences. Additionally, negative attitudes toward mentally ill patients can trigger an aversive affective response in non-patients, resulting in their withdrawal behaviors and avoidance. In both cases, putting oneself into the mind of mentally ill patients can induce personal distress rather than empathic concern, which in turn prevents care and support from reaching those with mental illness. This social neuroscience perspective may prompt new multi-level theoretical frameworks of health-related stigmas to guide stigma interventions, measurement, and public policy. It can also benefit our understanding of human social cognition, such as empathy, self-other similarity, and theory of mind, and their relationship to public health issues including stigma and lack of social support. Promising lines of future research are needed for improvement in public health as well as the theoretical richness of human social cognition.

## Data Availability Statement

The raw data supporting the conclusions of this article will be made available by the authors, without undue reservation.

## Ethics Statement

The studies involving human participants were reviewed and approved by Institutional Review Board of Seoul National University Hospital. The patients/participants provided their written informed consent to participate in this study.

## Author Contributions

W-GS developed the study concept. W-GS, HK, and JD developed the experimental task and contributed to the study design. Analyses were conducted by W-GS with guidance from C-WW and WJ. C-WW and WJ contributed to the interpretation of data. W-GS and JD wrote the article. JK managed the whole procedure of this study. All authors contributed to the article and approved the submitted version.

## Conflict of Interest

The authors declare that the research was conducted in the absence of any commercial or financial relationships that could be construed as a potential conflict of interest.

## Footnotes

^1^http://marsbar.sourceforge.net/
